# Were small businesses more likely to permanently close in the pandemic?

**DOI:** 10.1007/s11187-022-00662-1

**Published:** 2022-08-08

**Authors:** Robert Fairlie, Frank M. Fossen, Reid Johnsen, Gentian Droboniku

**Affiliations:** 1 Department of Economics, University of California, Santa Cruz, CA USA; 2 Stanford University, Stanford, CA USA; 3 NBER, Cambridge, MA USA; 4 Department of Economics, University of Nevada, Reno, NV USA; 5 IZA, Bonn, Germany; 6 California Department of Tax and Fee Administration, Sacramento, CA USA

**Keywords:** Small business, Entrepreneurship, Survival, Closures, Self-employment, COVID-19, Coronavirus, Pandemic, Shelter-in-place restrictions, Social distancing restrictions, L26, H25, I18

## Abstract

Previous estimates indicate that COVID-19 led to a large drop in the number of operating businesses operating early in the pandemic, but surprisingly little is known on whether these shutdowns turned into permanent closures and whether small businesses were disproportionately hit. This paper provides the first analysis of permanent business closures using confidential administrative firm-level panel data covering the universe of businesses filing sales taxes from the California Department of Tax and Fee Administration. We find large increases in closure rates in the first two quarters of 2020, but a strong reversal of this trend in the third quarter of 2020. The increase in closures rates in the first two quarters of the pandemic was substantially larger for small businesses than large businesses, but the rebound in the third quarter was also larger. The disproportionate closing of small businesses led to a sharp concentration of market share among larger businesses as indicated by the Herfindahl–Hirschman Index with only a partial reversal after the initial increase. The findings highlight the fragility of small businesses during a large adverse shock and the consequences for the competitiveness of markets.

## Abstract (plain language summary)

Small businesses were more likely to close permanently during the early stages of the COVID-19 pandemic than large businesses. Although they rebounded strongly thereafter, market concentration remains higher than before the pandemic. We obtain these results from analyzing administrative firm-level data covering all businesses filing sales taxes in California. Our analysis contributes to research by demonstrating the fragility of small businesses during a crisis relative to large businesses. Our findings imply that small businesses may need additional support given the trend toward purchases from large online retailers.

## Introduction

The coronavirus pandemic led to unprecedented, widespread shutdowns of businesses in the USA and around the world. Stores, restaurants, factories, professional offices, and many other businesses shut down in the first few months due to policy mandates, downward demand shifts, health concerns, or other factors. A small but rapidly growing literature documents these impacts. For example, Fairlie (2020) finds that the number of active business owners in the USA plummeted from 15.0 million in February 2020 to 11.7 million in April 2020 and only partially rebounded by June. Losses to small business revenues and sales were also found to be large in the early stages of the pandemic with estimates ranging from 30 to 50% (Bloom et al., 2021; Fairlie & Fossen, 2022; Kim et al., 2020), and owners’ demand expectations were one-third lower than before the crisis (Balla-Elliott et al., 2022). Examining financial account data, Farrell et al. (2020) find that as early as by the end of March 2020, cash balances were 12% lower for all firms.

The emerging literature on the effects of COVID-19, however, has three major limitations. First, it provides limited information on the impacts on small businesses. There is a debate in the literature on whether small firms have a disadvantage during recessions in comparison to large firms due to their fragility or an advantage due to their flexibility, and empirical results are mixed. While Moscarini and Postel-Vinay (2012) find relatively stronger growth performance of small employers relative to large employers during economic downturns, Fort et al. (2013) report that small firms—both young and old, and especially small and young firms—are more sensitive to local cyclical shocks than large firms in the USA. Bartz and Winkler (2016) find that young firms, not small firms in general, were disproportionately negatively affected by the 2009 financial crisis in Germany.^1^ However, the COVID-induced recession is very different from previous recessions; for example, no previous recession experienced a drop in gross domestic product (GDP) of 31% in one quarter and then rebounded the next quarter with an increase of 34% (U.S. Bureau of Economic Analysis 2022). As this recession was triggered by an exogenous health crisis, unlike other cyclical economic downturns that may be related to endogenous creative destruction (Schumpeter 1934), it provides an opportunity to study the resilience of small versus large businesses toward a large, aggregate negative shock.

Second, there is very little evidence on permanent closures (Crane et al., 2022). Because of the difficulty in finding timely panel data and measuring long-term business closures, the important question of whether small businesses permanently closed in disproportionate numbers in the pandemic remains unanswered in the literature. Federal government sources of data are often not released quickly enough and the approval processes for gaining access to these confidential microdata sources are too slow to study permanent business closures in the pandemic.^2^ Third, following from the aforementioned limitations, the empirical literature is silent on the effects of permanent small business closures on the competitiveness of the market.

These gaps in the literature are important because small businesses might have been especially devastated by the shutdowns during the pandemic due to lost revenues, limited cash reserves, and continuing expenses.^3^ Many of the early-stage shutdowns of small businesses might have turned into permanent closures. Small businesses likely had less ability to quickly adjust to changes in regulations and demand when the pandemic hit, and may have had less ability to obtain financing needed for adjustments. Customers also might have felt fewer health concerns shopping in large retailers instead of small shops. If these permanent closures and consumer shifts disproportionately hit small businesses, then market share will be concentrated among fewer retailers lessening overall competition. Additionally, there is a concern that consumers shifted away from purchasing goods from small brick-and-mortar businesses to online retailers and large retailers with an online presence.

In this paper, we use firm-level panel data to provide the first examination of permanent closures among small businesses in the pandemic using government administrative microdata. We analyze business closures using administrative microdata from the California Department of Tax and Fee Administration (CDTFA) that cover the universe of businesses with taxable sales in the state. Our study is the first to use a similar measure as the Census and Bureau of Labor Statistics (BLS) definition of permanent business closure that requires a full year of no operations using administrative records. We address three key questions about the performance of small businesses in the pandemic. First, we explore what percentage of businesses with taxable sales closed in the first few quarters of the COVID-19 pandemic. Second, we examine whether business closure rates differ by initial business size. In particular, we test the hypothesis that small businesses disproportionately closed during the pandemic and identify how much of the differential was due to COVID-19 by adjusting for trend and seasonality. Third, we examine the resulting effects on market share concentration.

Using CDTFA administrative microdata, we find large increases in closures rates in the first two quarters of 2020, but a strong reversal of this trend in the third quarter of 2020. The increase in closures rates in the pandemic was substantially larger for small businesses than large businesses, but the rebound was also larger. The disproportionate closing of small businesses led to a concentration of market share among larger businesses. The Herfindahl–Hirschman Index (HHI) across all retail businesses in California increased markedly from the third quarter of 2019 to the third quarter of 2020 breaking a slow, steady upward trend over the prior 4 years. It only partially rebounded until the third quarter of 2021. These timely findings on permanent business closures among small businesses are crucial to adjust and calibrate adequately targeted policy responses supporting small businesses and their owners and employees. More generally, the results demonstrate the fragility of small businesses during a large adverse shock and the negative consequences for the competitiveness of markets.

## Previous studies of closures in the pandemic

Crane et al. (2022) provide an overview of the small number of papers studying business closures during the early stages of the COVID-19 pandemic and potential data sources. They note that the statistics on business closures provided by the BLS and the Census Bureau (see below) are released with a time lag that is too long to inform timely policy responses. Therefore, some researchers conduct their own surveys of firm samples (Bartik et al., 2020a) or analyze the narrower subset of closures that result in bankruptcy filings (Wang et al., 2020). The U.S. Census Bureau’s (2020) Small Business Pulse Survey provides valuable information about continuing small businesses (Mini, 2021) but is less useful to measure business closures because survey non-response is higher for businesses that have ceased operations (Buffington et al., 2021). Surveys conducted after the pandemic will likely find it very difficult to reach businesses that permanently closed and capture them in the data. The World Bank COVID-19 Impact Surveys, for example, measure whether businesses are (i) currently open, (ii) temporarily shut down (suspended services or production), or (iii) permanently closed using survey data.^4^ Survey response rates are likely dependent on the outcome of interest.

Substantial efforts have been devoted to tap non-traditional “real-time” data sources collected by private companies for their business purposes. Bartik et al. (2020b) use data from Homebase, a provider of clock-in/clock-out tracking software, and measure business shutdowns by firms stopping to report clock events. Kurmann et al. (2021) also use the Homebase data, but in order to distinguish between firm closures and sample churn, they additionally check whether business owners update their status in Google Places (the database behind Google Maps) or stop posting on Facebook if the authors can find a match. Rigobon et al. (2022) use data from Google Places in the retail and food service sectors in the downtown core of Ottawa/Gatineau. By comparing scraped data at two points in time, they identify business exits when a business is removed from the data. De Vaan et al. (2021) rely on foot traffic data from mobile applications provided by SafeGraph, focusing on service-oriented businesses. They identify closures by the change in the number of visitors. Yelp (2020) uses its online platform of business reviews to track business closures when owners update their Yelp pages. Chetty et al. (2020) rely on Womply, which aggregates transaction and revenue data from several credit card processors. Firms that report zero credit card revenue for 3 days in a row are counted as closures. These papers thereby focus on sectors in which time clocking or use of social media or credit cards is common. In sum, while these non-traditional data sources provide fast and important first impressions of developments, there are limits to their representativeness and ability to track permanent closures. To our knowledge, our study is the first to address these challenges using administrative panel data covering the universe of businesses with taxable sales over the first three quarters of the pandemic.

## Measuring closures, data, and methods

### Census and BLS approach to measuring closures

The U.S. Census Bureau (2021) and U.S. Bureau of Labor Statistics (2021) provide measures of business or establishment closures. In both the Census and BLS, published numbers of closures are measured over a 1-year window. The Census Bureau’s Business Dynamics Series measures closures by establishments that have positive employment in the first quarter of the initial year and zero employment in the first quarter of the subsequent year. The first quarter of each year is used because of the timing of employment information (i.e., March 12 payroll).^5^ The BLS publishes information from the Business Employer Dynamics on annual survival rates for establishments with employees. They also report the total number of closures each quarter.^6^ The BLS has conducted analyses on different time frames for measuring closures (Sadeghi, 2008).^7^

### Measuring closures with administrative tax panel microdata

The panel microdata used here are restricted access from the CDTFA. The microdata consist of sales and use tax (SUT) returns and capture all taxable sales for the universe of businesses in California. Using these panel data, we track taxable sales over time in each quarter for the same business. To define business closures, we identify businesses that had taxable sales in one quarter but had no taxable sales in the next four consecutive quarters. If a business has no taxable sales over the next year, then we assume that the business has permanently closed. If a business has any taxable sales in a quarter, then it is not closed. It is rare for a business to come back after four quarters of no taxable sales and thus we approximate by assuming that this is a permanent closure. The use of a four-consecutive quarter (i.e., full year) closure measure allows us to examine closures through the third quarter of the pandemic. We use quarterly data through 2021Q2 to capture closures for up to 2020Q3 (i.e., the last quarter a business reported sales was in 2020Q2).

Several issues arise in using the CDTFA administrative data and taking our approach. First, not all SUT accounts file on a quarterly basis. Some small accounts (with less than $1,200 in annual tax liability) file annually in either June or December, resulting in skewed closure rates in Q2 and Q4 of each year. Further, as some of these small taxpayers grow, they may be required to change from annual to quarterly filing. We remove from our study these small accounts that at any point in time filed on an annual basis. We also aggregate monthly filers up to the quarter. Second, SUT accounts do not necessarily correspond 1:1 to businesses. A single business can operate more than one SUT account. This is unusual and typically relevant only for large businesses. More commonly, multiple physical sites can file under a single SUT account. In these cases, the data do not reflect individual storefront closures, only account closures. Third, in a typical year, approximately 30% of filers report zero sales tax liability. These accounts are effectively closed, although their permit remains open. We treat these accounts as closed in quarters in which they file zero or negative tax liability.

### Robustness to alternative future windows for measuring closures

We define a business closure using a four-quarter forward looking window. A closure is defined as a business that does not show up with taxable sales the next four quarters. We do not check whether the business returns after these first subsequent quarters. We also examine closures using a shorter forward looking window, and define a closure with a two-quarter forward looking window. The patterns over time look similar to those for our four-quarter forward looking definition, which is reassuring. The shorter forward looking time period increases the likelihood that we will mistakenly define a business closure when that business comes back in a future quarter. We follow the BLS and Census definitions that focus on a four-quarter or full-year window.

### Regression specifications

To adjust for pre-pandemic time trends, allow for seasonal (quarter) fixed effects, and provide a direct estimate of the impact of the pandemic, we estimate the following regression equation for the probability of a business closure:

3.1 $${Y}_{it}=\alpha +\sum\nolimits_{s=1}^{3}{\gamma }_{s}{\mathrm{COVID}}_{st}+{\beta }^{^{\prime}}{X}_{it}+\lambda t+\sum\nolimits_{s=1}^{3}{\pi }_{s}{\mathrm{quarter}}_{st}+{\varepsilon }_{it},$$

where ${Y}_{it}$ is the closure for business *i* in quarter *t*, ${\mathrm{COVID}}_{st}$ are the three dummy variables for the quarters *s* of the three post-COVID quarters in our data with information on closure (2020Q1–2020Q3), ${X}_{it}$ includes business characteristics, $\lambda$ is the slope of a linear time trend (with *t* set to zero at 2019Q4 and increasing by 0.25 each quarter), ${\mathrm{quarter}}_{st}$ is a set of quarter of the year dummy variables to control for seasonality, and ${\varepsilon }_{it}$ is the error term. The analysis sample period covers 22 quarters, with nineteen pre-COVID quarters (2015Q2–2019Q4) and three post-COVID quarters. Technically, the first quarter of 2020 includes the months of January and February which were prior to social distancing restrictions, and March only partly captures those restrictions, but as we show below large negative effects of the pandemic show up on gross domestic product (GDP) growth in 2020Q1.

The parameters of interest are the ${\gamma }_{s}$, which capture the estimates of COVID-19 effects on business closures relative to pre-pandemic levels after controlling for a time trend and seasonality. The equation will be estimated with OLS and robust standard errors. Using a linear probability model has the advantage over probit or logit of direct interpretability of the estimated coefficients of the interaction terms we introduce below. Additionally, with more than 7 million observations in the administrative data, any losses in efficiency are not a concern.

We also estimate the effects of COVID-19 on small businesses relative to large businesses. We define small businesses as those with average quarterly taxable sales annualized to less than $500,000, and calculate this measure over the previous four quarters. We estimate the following equation:

3.2 $${Y}_{it}=\alpha +{{\alpha }^{\mathrm{Sm}} \mathrm{Small}}_{i}+\sum\nolimits_{s=1}^{3}{\gamma }_{s}{\mathrm{COVID}}_{st}+\sum\nolimits_{s=1}^{3}{\gamma }_{s}^{\mathrm{Sm}}{\mathrm{COVID}}_{st}\times {\mathrm{Small}}_{i}+{\beta }^{^{\prime}}{X}_{it}+\lambda t+{\lambda }^{\mathrm{Sm}} t\times {\mathrm{Small}}_{i}+\sum\nolimits_{s=1}^{3}{\pi }_{s}{\mathrm{quarter}}_{st}+\sum\nolimits_{s=1}^{3}{\pi }_{s}^{\mathrm{Sm}}{\mathrm{quarter}}_{st}\times {\mathrm{Small}}_{i}+{\varepsilon }_{it},$$

where Small is a dummy variable for small businesses. In this specification, the parameters of interest are ${\gamma }_{s}^{\mathrm{Sm}}$, which capture the estimates of potential disproportionate effects of COVID-19 on small businesses relative to large businesses in the sense of a difference-in-differences estimator. Relative effects on business closures are estimated for each post-pandemic quarter. Pre-pandemic trends in business closures rates as well as seasonality are also allowed to differ by baseline size of the business. These estimates do not estimate causal effects of a policy change but instead capture more exploratory estimates of changes in business dynamics during the COVID-19 pandemic.

## Results

### Graphical evidence

**Fig. 1:**
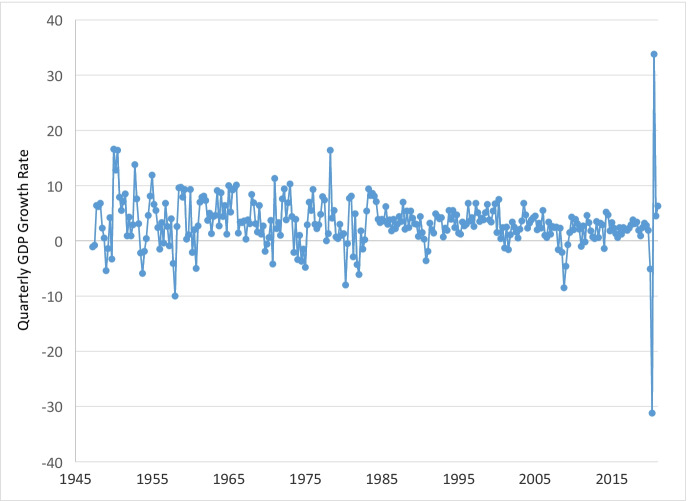
Quarterly gross domestic product growth rate, 1947Q2–2021Q1

**Fig. 2:**
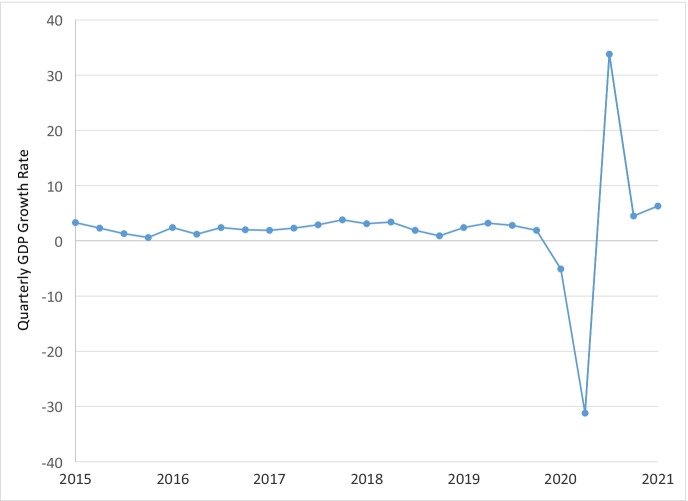
Quarterly gross domestic product growth rate, 2015Q1–2021Q1

Before examining business closure rates using the universe of businesses included in the CDTFA administrative data, we present evidence on the economic disruption caused by the pandemic. Figure 1 displays quarterly GDP growth from 1947Q2 to 2021Q1.^8^ Over the numerous recessions in the second half of the twentieth century and the first two decades of the twenty-first century, there has never been such a large quarter-to-quarter change in GDP as in the first full quarter in the pandemic, 2020Q2. GDP fell by 31.2% in 2020Q2 (Fig. 2 focuses on the period from 2005Q1 to 2021Q1). GDP also fell by 5.1% in 2020Q1. The next largest drops in GDP were by − 10.0% in 1958Q1, − 8.5% in 2008Q3 (Great Recession), and − 8.0% in 1980Q2. Furthermore, only three additional quarters over this time period experienced larger drops than in 2020Q1. The pandemic created an extremely severe but short recession. GDP reversed course quickly and grew by 33.8% in 2020Q3. The NBER officially dates the pandemic-induced recession as occurring from February 2020 (peak) to April 2020 (trough). As shown in Figs. 1 and 2, both 2020Q1 and 2020Q2 were affected severely by the beginning of the pandemic.

**Fig. 3:**
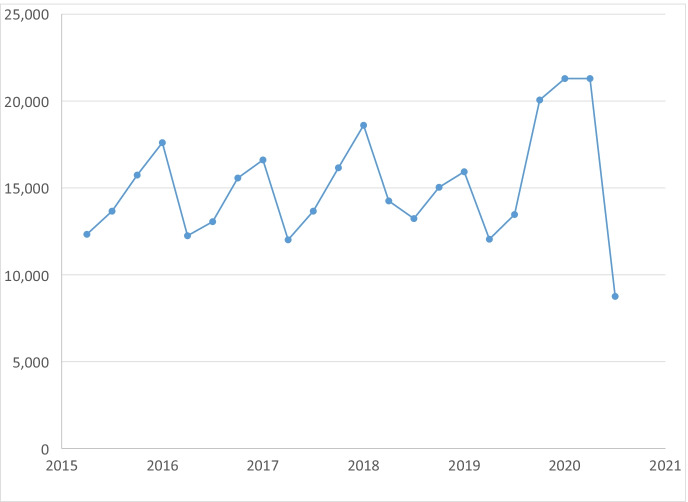
Total number of business closures per quarter, 2015Q2 to 2020Q3

Having established overall effects on the US economy, we turn to examining the total number of business closures before the pandemic and in the pandemic.^9^ Figure 3 displays the total number of business closures in California by quarter from 2015Q2 to 2020Q3. As noted above, we check for four consecutive quarters to make sure the business is not active before defining a closure.^10^ The number of business closures increased substantially in 2020Q1 and remained high in 2020Q2, capturing the early effects of COVID-19.^11^ After those initial increases, however, the number of business closures dropped back down to below pre-pandemic levels in 2020Q3. There is a strong seasonality component to business closures that makes it difficult to identify changes in the pandemic. Focusing on pre-pandemic data (i.e., 2015 to 2019), the average number of business closures over this time period is 17,200 in Q1, 12,600 in Q2, 13,400 in Q3, and 16,500 in Q4. Given that Q2 is typically a lower quarter for the number of closures, the jump in 2020Q2 is especially large relative to pre-pandemic levels. We return to these issues in the regressions below where we control directly for season/quarter dummies.

**Fig. 4:**
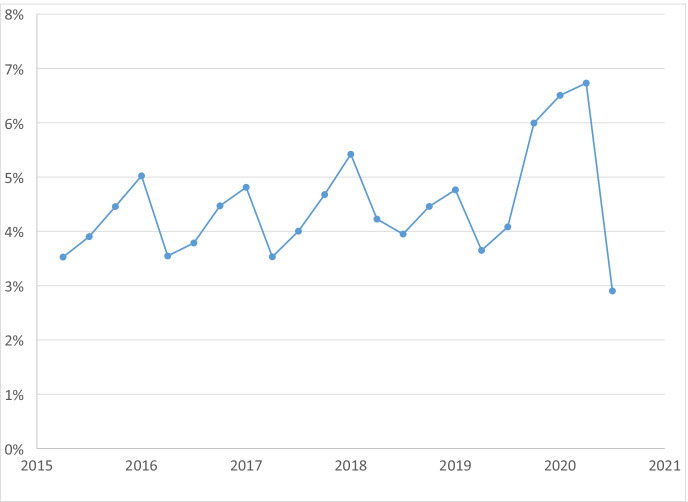
Total business quarterly closure rate, 2015Q2 to 2020Q3

We next focus on business closure rates, which is important for comparing small to large businesses.^12^ To start, Fig. 4 displays business closure rates by quarter from 2015Q2 to 2020Q3. The pandemic led to large increases in closure rates. Focusing on the YOY change, the closure rate was 3.7% in 2019Q2 and jumped to 6.7% in 2020Q2. From 2019Q1 to 2020Q1, the closure rate increased from 4.8 to 6.5%. After the initial shakeout of these two quarters in the pandemic, the closure rate in the third quarter dropped precipitously. Focusing on the YOY comparison, the closure rate in 2020Q3 was 2.9%, which was substantially lower than the closure rate of 4.1% in 2019Q3. The drop in closure rates reflects the exceptionally strong rebound in the economy in 2020Q3 as shown in GDP trends reported in Fig. 2. Closure rates display strong seasonality patterns but no strong downward or upward trend prior to the pandemic.

**Fig. 5:**
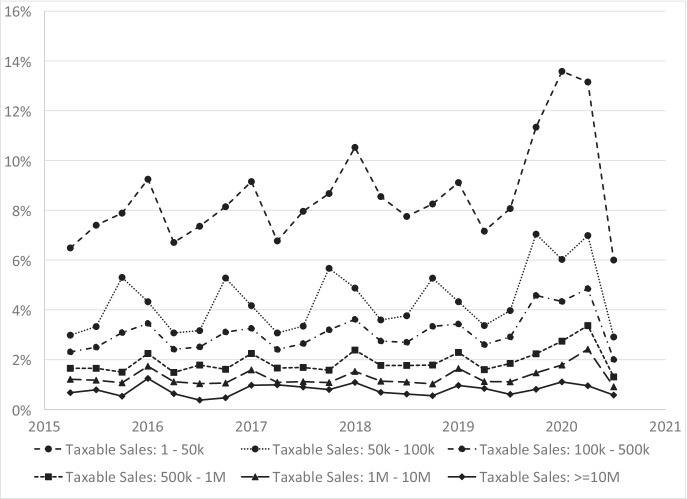
Quarterly closure rates by taxable sales size, 2015Q2 to 2020Q3

The large increase in the closure rate from 2019Q2 to 2020Q2, which captures the worst of the pandemic, might have been felt very differently by size of businesses. Figure 5 displays closure rates for several size categories of businesses based on the level of taxable sales in the previous quarter. There is a clear pattern of lower closure rates with initial size of the business which holds for all time periods. Closure rates are much higher for the small size classes than for the large size classes, and this differential holds prior the pandemic and after the pandemic. Another clear pattern is that all business sizes experienced an increase in closure rates in the pandemic. The jumps in closure rates disrupted the seasonality patterns. Another finding is that there does not appear to be a strong upward or downward trend prior to the pandemic in closure rates for any size class.

**Fig. 6:**
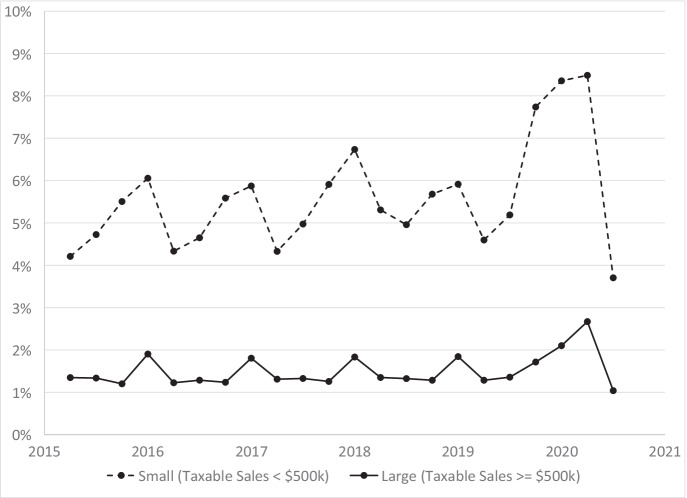
Quarterly closure rates for small and large businesses, 2015Q2 to 2020Q3

To focus on the experience of small businesses relative to large businesses, we simplify by collapsing categories. We also revise the definition of categories slightly. Instead of using taxable sales in the previous quarter to define the size class, we use an average of the prior four quarters to determine if a business is small or large. By averaging over the previous four quarters, we avoid some of the issues with COVID affecting which size class the business is in for calculating closure rates. Figure 6 displays closure rates for small businesses and large businesses from 2015Q2 to 2020Q3. We define small businesses as those with average quarterly taxable sales annualized to less than $500,000. Large businesses are those with $500,000 or more in annualized taxable sales over the previous 4 quarters. Closure rates are much higher for small businesses than for large businesses prior to the pandemic and in the pandemic. On average, 5.4% of small businesses closed each quarter from 2015Q1 to 2019Q4. In comparison, an average of 1.4% of large businesses closed each quarter during the same time period.

For both small and large businesses, there is a strong seasonality component with the highest closure rates happening in the first quarter of each year before the pandemic. COVID affected both small and large businesses. Closure rates increased from 4.6% in 2019Q2 to 8.5% in 2020Q2 for small businesses and increased from 1.3% in 2019Q2 to 2.7% in 2020Q2 for large businesses. The increase in closure rates is much larger for small businesses than for large businesses.

### Regression estimates

To adjust for pre-pandemic time trends, allow for seasonal fixed effects, and provide a direct estimate of the impact of the pandemic, we estimate Eq. 3.1 for the probability of a business closure. Estimates are reported in Table 1. We start with specification 1, which is the base model. After controlling for the time trend and seasonal effects, we find that COVID-19 had a large effect on business closures. In 2020Q2, business closures were 2.7 percentage points higher due to COVID. The coefficient implies a very large relative effect: the average closure rate in 2019 was 4.6%. The early effects of the pandemic were also felt in 2020Q1. Our regression model indicates an increase of 1.2 percentage points in the closure probability. By the summer/early fall of 2020 (2020Q3), we find that closure rates are lower than expected (i.e., no COVID disruption). The coefficient estimate for the 2020Q3 dummy indicator is negative implying that they are 1.4 percentage points lower than where they were expected to be.

**Table 1:** Probability of business closure—regression results

	(1)	(2)	(3)	(4)
Small			0.051***	0.051***
			(0.000)	(0.001)
2020Q1	0.012***	0.012***	0.002**	0.002*
	(0.000)	(0.000)	(0.001)	(0.001)
2020Q1 × small			0.015***	0.015***
			(0.001)	(0.001)
2020Q2	0.027***	0.026***	0.013***	0.013***
	(0.000)	(0.000)	(0.001)	(0.001)
2020Q2 × small			0.020***	0.020***
			(0.001)	(0.001)
2020Q3	− 0.014***	− 0.014***	− 0.004***	− 0.004***
	(0.000)	(0.000)	(0.001)	(0.001)
2020Q3 × small			− 0.015***	− 0.015***
			(0.001)	(0.001)
Time trend	0.001***	0.001***	0.000***	0.000
	(0.000)	(0.000)	(0.000)	(0.000)
Time trend × small			0.002***	0.002***
			(0.000)	(0.000)
Quarter 1	0.002***	0.002***	0.005***	0.005***
	(0.000)	(0.000)	(0.000)	(0.000)
Quarter 1 × small			− 0.004***	− 0.004***
			(0.001)	(0.001)
Quarter 2	− 0.010***	− 0.011***	− 0.000	− 0.000
	(0.000)	(0.000)	(0.000)	(0.000)
Quarter 2 × small			− 0.014***	− 0.014***
			(0.001)	(0.001)
Quarter 3	− 0.008***	− 0.008***	− 0.000	− 0.000
	(0.000)	(0.000)	(0.000)	(0.000)
Quarter 3 × small			− 0.011***	− 0.011***
			(0.001)	(0.001)
Pre 2019Q1		− 0.001**		− 0.001*
		(0.000)		(0.001)
Pre 2019Q1 × small			0.000	
				(0.001)
Constant	0.051***	0.051***	0.014***	0.014***
	(0.000)	(0.000)	(0.000)	(0.000)
Observations	7,441,320	7,441,320	7,441,320	7,441,320
*R*^2^	0.002	0.002	0.010	0.010

The data are the universe of businesses with taxable sales in California in each quarter from the California Department of Tax and Fee Administration (CDTFA). A closure is defined as a business that does not show up with taxable sales the next four quarters. Small (large) businesses are defined as those with average quarterly taxable sales annualized to less (more) than $500,000, which is calculated over the previous four quarters. Robust standard errors in parentheses. *p < 0.1; **p < 0.05; ***p < 0.01

As expected from the figures above, we do not find a strong pre-pandemic trend in closure rates over time. The coefficient estimate indicates an increase of only 0.1 percentage points each year. The coefficients do show a strong seasonality component with the highest closure rates in the first and fourth quarters and the lowest closure rates in the second and third quarters. All of the coefficients in these models are precisely estimated using the California administrative data with more than 7 million observations.

In specification 1, we compare the post-COVID effects to a pre-pandemic time period that encompasses the entire time period from 2015Q2 to 2019Q4. Our coefficient estimates of post-COVID quarters implicitly make comparisons to the average closure rate over this entire time period after adjusting for the linear time trend and seasonality. In specification 2, we alter the model to focus the comparison on 2019, which is the last year prior to the pandemic, by including a dummy variable that is one in any quarter prior to 2019Q1. We find essentially the same estimates of the effects of COVID on 2020 closure rates as noted above. We find an increase of 2.6 percentage points in the closure probability in 2020Q2. We also find an increase of 1.2 percentage points in 2020Q1, and a decrease of 1.4 percentage points in 2020Q3.

Overall, the regression estimates indicate that COVID-19 had a large effect on business closures in the first two quarters of 2020 and especially the second quarter of 2020. Business closure rates increased substantially in this quarter regardless of whether they are compared to the year just prior to the start of the pandemic or to a longer pre-pandemic period. The adverse effects of COVID already show up in the first quarter of 2020, which is consistent with the drop in GDP in this quarter shown above. Finally, the effects of COVID on closure rates reversed by the third quarter of 2020 in the economic rebound.

We turn to exploring how small businesses fared in the pandemic relative to large businesses by estimating Eq. 3.2. The main coefficients of interest are the interactions between the small business dummy variable and the post-COVID quarter dummy variables. Specification 3 uses the entire 2015Q2 to 2019Q4 time period as the pre-pandemic comparison period whereas specification 4 compares to 2019 by including the pre 2019Q1 dummy. The results from both specifications are again very similar. Looking at the difference-in-difference (DID) estimate noted above to estimate COVID-19 effects, we find that small businesses experienced a 2.0 percentage point higher closure rate in 2020Q2 relative to large businesses due to COVID-19. Small business were also more negatively affected in 2020Q1 with a 1.5 percentage point higher increase in closure rates due to the pandemic.

The negative effects of the pandemic on closure rates among large businesses were large in the second quarter of 2020 but small in the first quarter of 2020. The coefficient estimates indicate that the effect of COVID-19 on large businesses was an increase of 1.3 percentage points in 2020Q2 and 0.2 percentage points in 2020Q1. The combination of these main effect coefficients and the DID coefficients indicates that small businesses experienced a total increase of the closure rate by 3.3 percentage points in 2020Q2 from COVID. In 2020Q1, the total effect on small business closure rates was an increase of 1.7 percentage points.

Although small businesses were hit harder in the first two quarters of the pandemic than large businesses, they experienced a larger rebound in closure rates. In 2020Q3, small business closure rates decreased by 1.5 percentage points more than large business closure rates. Combining main and DID estimates, we find that the total effect on small business closure rates was a rebound of 1.9 percentage points in 2020Q3.

### Market concentration

Did the disproportionate closing of small businesses in the pandemic lead to an increase in the concentration of taxable sales at large businesses in California? To explore this question, we measure changes over time in market concentration using the commonly used Herfindahl–Hirschman Index (HHI). The use of administrative data covering the universe of businesses in California with taxable sales is important for an accurate measure of the HHI. Truncated, censored or windsorized data would otherwise lead to problems that we do not have with our data. For every quarter from 2015Q1 to 2021Q3, we calculate

4.1 $$HHI={S}_{1}^{2}+{S}_{2}^{2}+{S}_{3}^{2}+\cdots {S}_{N}^{2}$$

where *N* is the total number of businesses and *S*_*i*_ is the market share percentage (of taxable sales) of firm *i*.^13^ We do not limit the HHI to a particular industry or regions within California to explore this question broadly. By taking this approach, we take into account that during the pandemic, consumers shifted away from some industries (e.g., accommodation, food services and drinking places, and arts, entertainment, and recreation) toward other industries (e.g., grocery stores, building materials, and garden equipment) (Fairlie & Fossen, 2022). Note that the broader the industry and the wider the geographical location included to calculate the HHI, the smaller the HHI will be due to the inclusion of more businesses with market shares. But, we are interested here in examining possible *changes* in the HHI over time using a consistent definition, in particular comparing periods before and after the start of the COVID-19 pandemic. The *level* of the HHI we calculate cannot be compared directly to industry-specific HHI measures and does not inform whether the retail market in California is concentrated or not in absolute terms. The quantitative interpretation of the HHI is the following: The HHI divided by 100 is the probability (in percent) that two dollars chosen at random among all dollars spent in California in a given quarter are spent at the same retailer. An increase in the HHI indicates an increasingly concentrated market.

We adjust for a legislation change in California that would otherwise complicate the comparison of the HHI over time. Due to California’s Marketplace Facilitators Act (Assembly Bill No. 147), several large online retailers began collecting use tax on behalf of smaller retailers that used their sales platforms beginning in 2019Q4. In many cases, these large retailers opened new SUT accounts for their marketplace sales. These accounts tend to report very high taxable sales. We manually removed 13 marketplace facilitator accounts before calculating the HHI to avoid any confounding effects. It is possible that there are more marketplace facilitator accounts, since they are not required to identify themselves as such.

The quarterly HHI series shows some seasonality with higher concentration in the fourth quarter of each year, which is presumably related to the holiday shopping period. To deseasonalize the HHI, we first regress the quarterly HHI values from 2015Q1 to 2019Q3 on dummies for Q2, Q3, and Q4 and a constant. We do not include the quarters potentially impacted by the pandemic in this regression to estimate seasonal effects. Then we use the estimated model to predict the residuals based on all quarters from 2015Q1 to 2021Q3 and add back the estimated constant. This provides a deseasonalized HHI series which equals the observed HHI in each first quarter from 2015 to 2019.

**Fig. 7:**
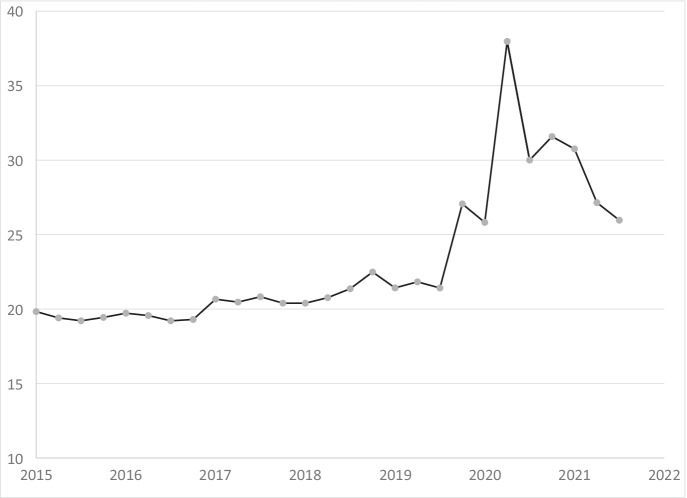
Herfindahl–Hirschman index for taxable sales, 2015Q1 to 2021Q3. Notes: Seasonally adjusted Herfindahl–Hirschman Index for all businesses with taxable sales in California after marketplace facilitators have been removed

Figure 7 plots the development of the deseasonalized HHI in California over time. The figure shows that the HHI increased only slowly and mostly steadily from 19.8 in 2015Q1 to 21.4 in 2019Q3 before the pandemic. During the pandemic, however, the HHI increased to a maximum of 38.0 during the strictest lockdowns in 2020Q2 and then decreased to 26.0 in 2021Q3 (the last measurement we have), but remains far higher than before the pandemic.^14^ Thus, the results show a clear increase in market concentration during the pandemic with a partial recovery in the second and third quarters of 2021. The probability that two random dollars were spent at the same retailer in California within a quarter increased from 0.214% in 2019Q3 to 0.260% in 2021Q3, which corresponds to a relative increase of 21%. This increased concentration is consistent with the evidence we provide on closures of small businesses during the COVID-19 pandemic.

## Conclusions

Although it is well known that COVID-19 led to a massive shutdown of stores, restaurants, and other businesses in the second quarter of 2020, surprisingly little is known about whether these shutdowns turned into permanent closures. The difficulty is in having a long enough time window to look for whether a closure is permanent and having comprehensive firm-level panel microdata. Using confidential administrative microdata from the California Department of Tax and Fee Administration, we provide new evidence on permanent closures among the universe of businesses with taxable sales in California. We find large increases in closures rates in the first two quarters of 2020, but a strong reversal of this trend in the third quarter of 2020. Regression results that control for the continuation of pre-pandemic trends indicate that COVID led to an increase of 1.2 percentage points in closure rates in the first quarter of 2020 and an increase of 2.6 percentage points in closure rates in the second quarter of 2020. Both increases in closure rates were large compared to the base closure rate of 4.6% in 2019. The rebound in the third quarter was also strong at 1.4 percentage points higher than expectations.

The novel evidence presented here on the impacts of COVID-19 on permanent closures among small business clearly indicates large disproportionate losses in the pandemic. In the first quarter of 2020, large businesses experienced only a slight increase in closure rates (0.2 percentage points), whereas small businesses experienced a substantial increase in closure rates (1.7 percentage points). In the second quarter of 2020, large businesses experienced an increase in closure rates of 1.3 percentage points whereas small businesses experienced an increase in closure rates of 3.3 percentage points due to COVID-19. The rebound in closure rates in the third quarter of 2020, however, was stronger for small businesses relative to large businesses (1.9 percentage points compared with 0.4 percentage points). The disproportionate losses among small businesses led to a concentration of market share among larger businesses as evidenced by an increase of 21% in the Herfindahl–Hirschman Index across all businesses with taxable sales in California.

The large closure rates in the first and second quarters of 2020 are worrisome for the longer-term survival of small, local businesses throughout the country. Will additional government assistance for small businesses be needed to reverse the increased concentration of market power among large businesses and keep the economy competitive? The Paycheck Protection Program targeted more than $800 billion in loans to small businesses, but currently there is no plan for additional funds. Another problem facing small businesses is that the shift in consumer behavior toward online purchasing during the mandated social distancing restrictions in the pandemic is unlikely to fully reverse. Consumers have become more accustomed to purchasing goods and services online, and small businesses are at a disadvantage in online sales relative to large retailers and online retailers. Small businesses will need to adjust and this might be an area in which government aid could be targeted. Additionally, some states and local governments have promoted shopping small and local (e.g., California’s #ShopSafeShopLocal) but can these programs counteract the closures of small businesses during the pandemic?

Our results suggest that on average, the fragility of small businesses in comparison to large businesses outweighs their higher flexibility when facing a large aggregate negative shock such as a health crisis, which is consistent with Fort et al. (2013). More research is needed on why the negative impacts of COVID-19 fell disproportionately on small businesses. Overall, small businesses might have had less ability to quickly adjust to changes in regulations and demand when the pandemic hit. Due to high fixed costs and required knowledge, small businesses may have faced larger barriers to increasing their web presence, expanding takeout services or adding delivery services, and coping with uncertainty regarding liability during the health crisis. In particular, small businesses may have had less ability to obtain financing needed for adjustments, for example, for investing in online ordering and inventory management, due to lower liquidity reserves, less collateral, and higher uncertainty from the perspective of lenders and investors during the emerging economic crisis. Customers in general might have felt fewer health concerns shopping in large retailers instead of small shops. As more data become available, these will be important questions for future research.

## Appendix

**Table 2:** Quarterly Business Closure Rates, 2015Q2to 2020Q3

Year/Quarter	Total	Taxable Sales: 1 - 50k	Taxable Sales: 50k - 100k	Taxable Sales: 100k - 500k	Taxable Sales: 500k - 1M	Taxable Sales: 1M - 10M	Taxable Sales: >=10M	Large	Small
2015Q2	3.5%	6.5%	3.0%	2.3%	1.7%	1.2%	0.7%	1.3%	4.2%
2015Q3	3.9%	7.4%	3.3%	2.5%	1.7%	1.2%	0.8%	1.3%	4.7%
2015Q4	4.5%	7.9%	5.3%	3.1%	1.5%	1.1%	0.5%	1.2%	5.5%
2016Q1	5.0%	9.2%	4.3%	3.5%	2.2%	1.7%	1.2%	1.9%	6.1%
2016Q2	3.5%	6.7%	3.1%	2.4%	1.5%	1.1%	0.6%	1.2%	4.3%
2016Q3	3.8%	7.4%	3.2%	2.5%	1.8%	1.0%	0.4%	1.3%	4.6%
2016Q4	4.5%	8.1%	5.3%	3.1%	1.6%	1.1%	0.5%	1.2%	5.6%
2017Q1	4.8%	9.2%	4.2%	3.3%	2.2%	1.6%	1.0%	1.8%	5.9%
2017Q2	3.5%	6.8%	3.1%	2.4%	1.7%	1.1%	1.0%	1.3%	4.3%
2017Q3	4.0%	8.0%	3.3%	2.6%	1.7%	1.1%	0.9%	1.3%	5.0%
2017Q4	4.7%	8.7%	5.7%	3.2%	1.6%	1.1%	0.8%	1.3%	5.9%
2018Q1	5.4%	10.5%	4.9%	3.6%	2.4%	1.5%	1.1%	1.8%	6.7%
2018Q2	4.2%	8.6%	3.6%	2.7%	1.8%	1.1%	0.7%	1.4%	5.3%
2018Q3	4.0%	7.8%	3.8%	2.7%	1.8%	1.1%	0.6%	1.3%	5.0%
2018Q4	4.5%	8.3%	5.3%	3.3%	1.8%	1.0%	0.5%	1.3%	5.7%
2019Q1	4.8%	9.1%	4.3%	3.4%	2.3%	1.6%	1.0%	1.8%	5.9%
2019Q2	3.7%	7.2%	3.4%	2.6%	1.6%	1.1%	0.8%	1.3%	4.6%
2019Q3	4.1%	8.1%	4.0%	2.9%	1.8%	1.1%	0.6%	1.4%	5.2%
2019Q4	6.0%	11.3%	7.0%	4.6%	2.2%	1.5%	0.8%	1.7%	7.7%
2020Q1	6.5%	13.6%	6.0%	4.3%	2.7%	1.8%	1.1%	2.1%	8.4%
2020Q2	6.7%	13.2%	7.0%	4.9%	3.4%	2.4%	1.0%	2.7%	8.5%
2020Q3	2.9%	6.0%	2.9%	2.0%	1.3%	0.9%	0.6%	1.0%	3.7%

Notes: The data are the universe of businesses with taxable sales in California in each quarter from the California Department of Tax and Fee Administration (CDTFA). A closure is defined as a business that does not show up with taxable sales the next four quarters. Small (large) businesses are defined as those with average quarterly taxable sales annualized to less (more) than $500,000, which is calculated over the previous four quarters

## Footnotes

1 Similarly, previous research on the basic relationship between unemployment and entrepreneurship provides mixed results. Parker (2018) reviews the literature and cites many previous studies showing positive relationships, negative relationships, and zero relationships. See Fairlie (2013), for example, for evidence of a positive relationship between unemployment in local labor markets and business creation using time series and cross-sectional variation, and Fossen (2021) for consistent results at the individual level.

2 For example, the latest published US Census Bureau’s (2021) data only reports closures between 2018 and 2019.

3 Just prior to the pandemic when small business owners were asked what actions they would take if faced with a 2-month revenue loss, roughly half said they would use their own funds and 17% said they would close or sell the business (Mills et al. 2020).

4 https://openknowledge.worldbank.org/handle/10986/34636

5 https://www.census.gov/programs-surveys/susb/about/glossary.html

6 E.g., Chart 5 at https://www.bls.gov/bdm/entrepreneurship/entrepreneurship.htm

7 Three measures are compared: (i) four consecutive quarters in which there is zero employment in the third month, (ii) five consecutive quarters in which there is zero employment in the third month, and (iii) twelve consecutive months of zero employment.

8 The Bureau of Economic Analysis first reports quarterly GDP growth rates in 1947Q2.

9 Appendix Table 2 shows closure rates by quarter and business size.

10 For example, to define a business closure in 2020Q2, the business must have taxable sales in 2020Q1, but no taxable sales in 2020Q2, 2020Q3, 2020Q4, or 2021Q1.

11 There are two reasons why we might observe an increase in closures already in 2019Q4. First, about 10% of businesses usually file their sales taxes more than 30 days late. Thirty days after the 2019Q4 filing deadline was March 1, 2020. Late filers who closed in 2020 due to the pandemic may not have filed their 2019Q4 returns and therefore show up as closing in 2019Q4 although they really closed in 2020. Second, the Marketplace Facilitator Act (Assembly Bill No. 147) took effect in 2019Q4. Starting in this quarter, California required online selling platforms to collect use tax on behalf of third party sellers that sell on those platforms. This eliminated the requirement to file for some online sellers that sold exclusively through online marketplaces and were correctly collecting use tax prior to AB-147 since the marketplace facilitator collects the tax now.

12 We do not need to report DHS corrected closure rates that average the previous quarter and current quarter number of businesses because with microdata we can examine closure business by business.

13 As usual for the HHI, the shares are expressed as a percent, not a fraction, for example, 5 if a firm has 5% of taxable sales in an industry, not 0.05.

14 The observation that the HHI starts increasing in 2019Q4 already is consistent with the observed increase of closures attributed to 2019Q4 and is likely to be due to late tax filers who closed in 2020 due to the pandemic (see footnote 11).
